# Interaction Between Rumen Microbiota and Epithelial Mitochondrial Dynamics in Tibetan Sheep: Elucidating the Mechanism of Rumen Epithelial Energy Metabolism

**DOI:** 10.3390/biotech14020043

**Published:** 2025-06-05

**Authors:** Ying Xu, Yuzhu Sha, Xiaowei Chen, Qianling Chen, Xiu Liu, Yanyu He, Wei Huang, Yapeng He, Xu Gao

**Affiliations:** 1 Gansu Key Laboratory of Herbivorous Animal Biotechnology, College of Animal Science and Technology, Gansu Agricultural University, Lanzhou 730070, China; 18298402040@163.com (Y.X.); shayz@st.gsau.edu.cn (Y.S.); cxw20002022@163.com (X.C.); chenqianling223@163.com (Q.C.); 18294737108@163.com (W.H.); 18894448066@163.com (Y.H.); gx2049879994@163.com (X.G.); 2 School of Fundamental Sciences, Massey University, Palmerston North 4410, New Zealand; y.h@massey.ac.nz

**Keywords:** Tibetan sheep, rumen, microorganisms, mitochondria, energy metabolism

## Abstract

Investigating the functional interactions between rumen microbial fermentation and epithelial mitochondrial dynamics/energy metabolism in Tibetan sheep at different altitudes, this study examined ultrastructural changes in rumen epithelial tissues, expression levels of mitochondrial dynamics-related genes (fusion: *Mfn1*, *Mfn2*, *OPA1*, *Mic60*; fission: *Drp1*, *Fis1*, *MFF*), and ketogenesis pathway genes (*HMGS2*, *HMGCL*) in Tibetan sheep raised at three altitudes (TS 2500m, TS 3500m, TS 4500m). Correlation analysis was performed between rumen microbiota/metabolites and mitochondrial energy metabolism. Results: Ultrastructural variations were observed across altitudes. With increasing altitude, keratinized layer became more compact; desmosome connections between granular layer cells increased; mitochondrial quantity and distribution in spinous and basal layers increased. Mitochondrial dynamics regulation: Fission genes (*FIS1*, *DRP1*, *MFF*) showed significantly higher expression at TS 4500m (*p* < 0.01); fusion genes (*Mfn1*, *OPA1*) exhibited altitude-dependent upregulation. Energy metabolism markers: Pyruvate (PA) decreased significantly at TS 3500m/TS 4500m (*p* < 0.01); citrate (CA) increased with altitude; NAD^+^ peaked at TS 3500m but decreased significantly at TS 4500m (*p* < 0.01); Complex II (SDH) and Complex IV (CO) activities decreased at TS 4500m (*p* < 0.01). Ketogenesis pathway: β-hydroxybutyrate increased significantly with altitude (*p* < 0.01); acetoacetate peaked at TS 2500 m/TS 4500 m; *HMGCS2* expression exceeded *HMGCL*, showing altitude-dependent upregulation at TS 4500m (*p* < 0.01). Microbiome–metabolism correlations: Butyrivibrio_2 and Fibrobacter negatively correlated with *Mic60* (*p* < 0.01); Ruminococcaceae_NK4A214_Group positively correlated with *Mfn1/OPA1* (*p* < 0.05); WGCNA identified 17 metabolite modules, with MEturquoise module positively correlated with *DRP1/Mfn2/MFF* (*p* < 0.05). Conclusion: Altitude-induced ultrastructural adaptations in rumen epithelium correlate with mitochondrial dynamics stability and ketogenesis upregulation. Mitochondrial fission predominates at extreme altitudes, while microbiota–metabolite interactions suggest compensatory energy regulation mechanisms.

## 1. Introduction

Tibetan sheep is the oldest breed of sheep on the Tibetan Plateau, bred in the Tibetan Plateau region at an altitude of more than 2500 m, with an inventory of about 30 million sheep, which is the largest population of sheep in China and a major livestock and poultry genetic resource on the Tibetan Plateau. The Tibetan sheep is a unique sheep bred on the Tibetan Plateau formed through long-term natural and artificial selection in the alpine and low-oxygen habitats [1,2]; it has the characteristics of cold tolerance, low oxygen tolerance, roughage tolerance, and strong disease resistance, and is the main germplasm resource for the development of plateau animal husbandry. The rumen, as a unique digestive organ of ruminants, plays an important role in the host’s metabolism, immune function, and health [3]. The unique structure of the rumen epithelium influences the net utilization of nutrients and energy by the ruminant organism, and the symbiotic relationship between its microflora and its host relies heavily on a constant supply of nutrients from their diet. The rumen epithelium is a stratified squamous epithelium with a basal layer, a spiny layer, a granular layer, and a cuticle, each of which has its own unique structure and biological function [4]. The rumen epithelium has a number of important physiological functions including absorption, transport, and protection [5]. The rumen microbiota stimulates mitochondrial biogenesis through the secretion of short-chain fatty acids (SCFAs), such as butyrate, thereby enhancing energy metabolism [6,7]. Mitochondria are dynamic organelles, capable of constantly changing their shape through cleavage and fusion, and they can transition between a long interconnected network-like shape and a disjointed fragmented state, a process known as mitochondrial dynamics [8]. Under normal conditions, mitochondrial division and fusion are maintained in dynamic equilibrium as a means of maintaining normal mitochondrial morphology, distribution, and function [9]. Bean et al. [10] showed that cell lifespan can be prolonged and beneficial to the health of the organism by regulating mitochondrial dynamics, in which *Drp1* (Mitochondrial dynamin-related protein 1) is a key regulator of mitochondrial fission and an important factor in maintaining the balance of mitochondrial fusion and division [11]. In general, *Drp1* and *Fis1* (Mitochondrial fission protein 1) mediate mitochondrial fission [12,13,14]. As a double-membrane organelle, the complete fusion of mitochondria requires a combination of inner and outer membranes, with the fusion of its outer membrane mainly relying on the GTPase transmembrane protein, *Mfn1/2* (Mitochondrial fusion proteins) on the outer mitochondrial membrane, and the fusion of the inner membrane being taken care of by *OPA1* [15]. When cells lack *Mfn1/2*, fusion is not possible, whereas *OPA1* requires *Mfn1* for endosomal fusion [16], and increased *Mfn1* expression protects mitochondrial metabolic function [17]. *Mfn2* plays a key role in regulating cellular endoplasmic reticulum morphology and mitochondrial fusion. It has been shown that *Mic60* plays an important role in mitochondrial cristae organization [18], protein transport [19], mt DNA (mitochondrial DNA), transcription [20], ATP generation [21], and apoptosis [22].

Mitochondria are the main structure for cellular energy production and the main site of aerobic respiration, known as the “energy factory” of the cell, which not only participates in energy metabolism but also in apoptosis, signaling, cell proliferation and cellular metabolism, and other important life processes [23], and the realization of these functions relies on the regulation of energy metabolism-related proteins and products [24]. The TCA cycle is the main pathway for ATP production and is involved in the catabolism of sugars, proteins, and fats, in which citrate synthase plays a key regulatory role in the TCA cycle by catalyzing the condensation of acetyl CoA with oxaloacetate to form citric acid [25]. Reduced/oxidized nicotinamide adenine dinucleotide (NADH/NAD^+^) is a key cofactor in living organisms that plays an important role in biosynthesis, catabolism, and cellular energy transfer, and it has the ability to regulate energy metabolism, regulate the redox state of the cell, control carbon fluxes, and improve mitochondrial activity and other functions [26]. PA (Pyruvic acid), CA (Citric acid), and NADH/NAD^+^ (Nicotinamide adenine dinucleotide) are key enzymes in the aerobic oxidation pathway. The TCA cycle determines the ability of mitochondria to produce ATP. SDH (Succinate dehydrogenase) is the only enzyme complex involved in both mitochondrial oxidative phosphorylation and the tricarboxylic acid cycle [27]. Co (Cytochrome c oxidase) is the only cytochrome capable of transferring electrons to oxygen molecules [28,29], and it also assumes the function of electron transfer from cytochrome C to oxygen molecules, which is a key regulatory site for the oxidative capacity of mitochondria [30].

Mitochondria provide ATP to rumen epithelial cells through oxidative phosphorylation, supporting their metabolic activities (e.g., cell proliferation, ion transport, and digestive enzyme secretion) [31]. During rumen fermentation, the volatile fatty acids (VFAs) produced by microbes rely on mitochondrial oxidation for energy supply. Mitochondria participate in reactive oxygen species (ROS) generation and clearance, maintaining the antioxidant defense system of rumen epithelial cells to prevent oxidative damage caused by microbial metabolic products [2]. The mitochondrial density in the rumen epithelium of high-altitude sheep is significantly higher than that of lowland breeds, compensating for energy deficits under hypoxic conditions. Tibetan sheep carrying specific mitochondrial DNA haplotypes exhibit enhanced efficiency of oxidative phosphorylation [32]. Studies have demonstrated that ruminants inhabiting high-altitude regions (e.g., Alpine sheep and buffalo) generally exhibit higher mitochondrial densities compared to their lowland counterparts, though their adaptive mechanisms differ [31]. For instance, Alpine sheep rely on alterations in mitochondrial membrane lipid composition to enhance cold tolerance, whereas North American highland species prioritize upregulation of mitochondrial antioxidant enzyme expression [33]. For every 1000 m increase in altitude, mitochondrial number increases by approximately 15%, but this effect may be modulated by dietary factors and growth stage [34].

Ruminal ketogenesis in ruminants is a process in which butyric acid is converted in mitochondria to produce acetyl CoA, which is further converted to ketone bodies such as β-hydroxybutyric acid and acetoacetate. About 90% of the butyric acid is oxidized to produce ketone bodies, and the ketone bodies are dominated by β-hydroxybutyric acid [35]. β-Hydroxybutyric acid, which is less abundant in the animal body, is an important chemical in the healthy growth process of ruminants, and it participates in the growth and development of the animal organism as an energy substrate and signaling molecule, among other forms [35], and it plays an important role in the growth of the rumen epithelium of young ruminants, marking the development of its metabolic function [36]. Butyric acid is absorbed by the rumen epithelium and metabolized to ketogenic pathways by the acetyl CoA synthase family to generate acetyl CoA [37]. A portion of acetyl CoA enters the TCA (tricarboxylic acid) cycle to release substantial energy, while the remaining acetyl CoA in rumen epithelial cell mitochondria undergoes ketogenesis catalyzed by the key enzyme HMGCS (β-hydroxy-β-methylglutaryl-CoA synthase). This enzyme facilitates the condensation of acetyl CoA with acetoacetyl CoA to form HMG-CoA (β-hydroxy-β-methylglutaryl coenzyme A). Subsequently, HMGCL (β-hydroxy-β-methylglutaryl-CoA lyase) cleaves HMG-CoA to release acetoacetate. Most acetoacetate is further metabolized by β-hydroxybutyrate dehydrogenase into β-hydroxybutyrate, which ultimately enters the bloodstream. The ATP generated through this process provides approximately 80% of the energy required by rumen epithelial cells [38].

Therefore, based on the comparative analysis of the ultrastructure of the rumen epithelium, tissue-like mitochondrial dynamics, and genes related to the ketogenic pathway in Tibetan sheep at different altitudes, the present study was conducted to further analyze the correlation of rumen microbes and their metabolites with key enzymes and genes involved in mitochondrial energy metabolism. The aim was to obtain the characteristics of the changes in the rumen tissues and mitochondrial structure at different altitudes in Tibetan sheep as well as to reveal the Tibetan sheep rumen microbial flora and metabolites interacting with the host, providing a basis for plateau adaptation studies in Tibetan sheep to better manage the health and well-being of the whole animal.

## 2. Materials and Methods

### 2.1. Ethics Statement

All studies involving animals were carried out in accordance with the regulations for the Administration of Affairs Concerning Experimental Animal (Ministry of Science and Technology, China; revise in June 2004), and sample collection protocols were approved by the Livestock Care Committee of Gansu Agricultural University (Approval No. GSAU-EthAST-2021-24).

### 2.2. Experiment Design and Sample Collection

**Figure 1:**
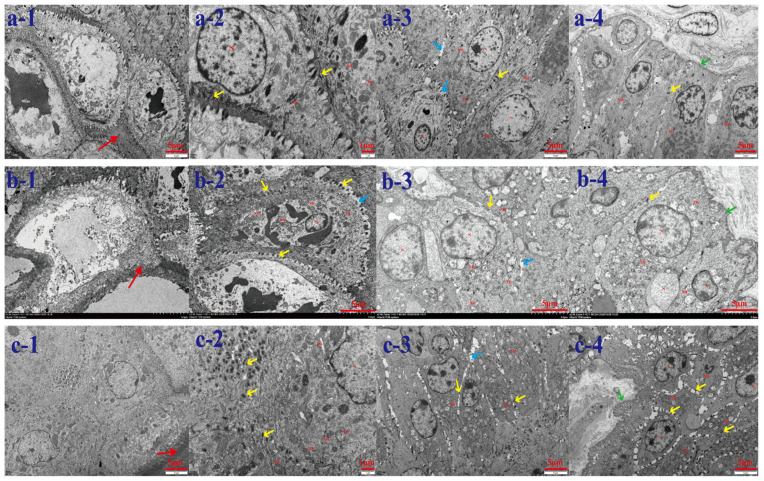
Ultrastructure of rumen epithelium of Tibetan sheep at different altitudes. (**a**) represents 2500m, (**b**) represents 3500m, (**c**) represents 4500m; 1–4 represent stratum corneum, stratum granulosum, stratum spinosum, and stratum basale, respectively. Red arrow: basal layer; yellow arrow: spinous layer; blue arrow: granular layer; green arrow: stratum corneum.

Plateau-type grazing Tibetan sheep were used as research subjects (around 3.5 years old, ♀, **n** = 6 sheep/group), and all experimental sheep were under local traditional natural grazing management without any supplemental feeding. Samples of Tibetan sheep were collected in August 2020 at different altitudes on the Tibetan Plateau (Figure 1), namely Zhuoni (TS 2500m), Haiyan (TS 3500m), and Yushu (TS 4500m). Before morning grazing, the samples were slaughtered according to the ethical approval of the Ethics Committee, and the rumen organs were removed within 10 min after slaughter, and a large piece of rumen epithelial tissue (abdominal pouch area) was clipped and rinsed well with saline. A small portion of the sample was quickly frozen in a freezing tube for subsequent transcriptome sequencing and enzyme assays, and the other portion was trimmed to 1 cm^3^ and fixed in 2.5% glutaraldehyde for transmission electron microscopy sectioning, while the rumen contents were collected and preserved in liquid nitrogen for the determination of 16S rRNA and VFAs.

### 2.3. Extraction of Total RNA from Rumen Epithelium

RNA Extraction using Trizol Reagent (DP762-T1C) from Tibetan Sheep Rumen Epithelial Tissue. Retrieve frozen rumen epithelial tissue samples and immediately transfer them to a liquid nitrogen-cooled mortar for grinding. Transfer the powdered sample to a 1.5 mL centrifuge tube, add 1 mL RNA isolation buffer (RNA Isolater), and incubate at room temperature for 5 min. Centrifuge at 12,000× *g* and 4 °C for 5 min. Carefully aspirate the supernatant into a new tube. Add 200 μL chloroform, vortex vigorously for 15 s to form an emulsion, and incubate at 4 °C for 5 min. Centrifuge at 12,000× *g* and 4 °C for 15 min. Transfer 500 μL of the upper aqueous phase to a fresh tube. Add an equal volume of ice-cold isopropanol, invert gently to mix, and incubate at 4 °C for 10 min. Centrifuge at 12,000× *g* and 4 °C for 5 min. Discard the supernatant and air-dry the pellet in a laminar flow hood for 3 min (avoid over-drying). Resuspend the pellet in 50 μL DEPC-treated water (ultrapure water) until fully dissolved. Measure RNA concentration and purity using a NanoDrop 2000 (Thermo Fisher Scientific, Wilmington, DE, USA). Assess RNA integrity via Agilent Bioanalyzer 2100 (Agilent Technologies, Santa Clara, CA, USA) with the RNA Nano 6000 Assay Kit.

### 2.4. Observations on the Ultrastructure of Rumen Epithelium

#### 2.4.1. Fixation and Dehydration

Reticulorumen tissue was fixed in 4% paraformaldehyde solution for 24 h. Subsequently, the fixed samples underwent graded dehydration in the following ethanol series: (1) 50% ethanol, 2 h; (2) 75% ethanol, 2 h (overnight if necessary); (3) 85% ethanol, 2 h; (4) 95% ethanol, 1 h.

#### 2.4.2. Clearing and Embedding

After briefly draining residual ethanol, the dehydrated tissue was immersed in xylene for clearing until transparent (approximately 15 min). The cleared samples were then embedded into molten paraffin at 60 °C for 2 h, followed by paraffin block formation.

#### 2.4.3. Sectioning and Staining

Sectioning: Paraffin blocks were mounted in a microtome and sectioned at 3 μm thickness. The sections were floated on 45 °C distilled water, mounted on slides, and dried at 60 °C.

#### 2.4.4. Hematoxylin–Eosin Staining

(1) Xylene I dewaxing, 10–15 min; (2) Xylene II dewaxing, 1–2 min; (3) Xylene: absolute ethanol (1:1), 1–2 min; (4) absolute ethanol, 1–2 min; (5) descending alcohol gradient hydration (95%, 85%, 75%, and 50% ethanol, each 1–2 min); (6) distilled water rinsing, 1–2 min; (7) hematoxylin staining, 10–15 min; (8) distilled water rinsing to remove excess dye; (9) acid alcohol differentiation, 30–60 s; (10) tap water rinsing for bluing, 15–20 min; (11) distilled water rinsing, 1–2 min; (12) ascending alcohol gradient dehydration (50%, 75%, and 85% ethanol, each 1–2 min); (13) eosin staining, 2–3 min; (14) ascending alcohol gradient dehydration (75%, 85%, 95%, and absolute ethanol, each 1–2 min); (15) Xylene I and II clearing, total 15 min. Slides were mounted with neutral resin, air-dried, and imaged under a microscope.

#### 2.4.5. Ultrastructural Analysis

Using the Slide Viewer system, ultrastructural features of the rumen epithelium—including stratum corneum, stratum granulosum, stratum spinosum, and stratum basale—were analyzed. Key observations included mitochondrial distribution/architecture and desmosomal connections between cells.

### 2.5. Studies of Mitochondrial Dynamics and Function

The expression of mitochondrial dynamics-related genes (split: *Drp1*, *Fis1*, *MFF*; fusion: *Mfn1*, *Mfn2*, *OPA1*, *Mic60*) and ketogenic pathway-related genes (*HMGS2*, *HMGCL*) was determined in the rumen epithelial tissue samples of Tibetan sheep grazing at different altitudes. Fluorescence quantification of related genes and internal reference genes was performed using an Applied Biosystems Q6 real-time fluorescence quantitative PCR instrument. Reaction conditions: Pre-denaturation at 95 °C for 30 s; cycling reaction at 95 °C for 10 s, 60 °C for 30 s, 40 cycles; lysis curve (95 °C for 15 s, 60 °C for 60 s, 95 °C for 15 s). Reaction system: 20 uL system containing 2 × ChamQ Universal SYBR qPCR Master Mix, cDNA template, and upstream and downstream primers. β-actin was used as the internal reference gene for correction, and the data were analyzed by the method of 2^−∆∆CT^. Gene primer information is shown in Table 1.

The key enzymes of the mitochondrial tricarboxylic acid cycle and oxidative phosphorylation, as well as the ketogenic pathway, were measured by the assay kits of Suzhou Keming Biotechnology Co. The key enzymes of the mitochondrial tricarboxylic acid cycle and oxidative phosphorylation, as well as the ketogenic pathway, were pyruvic acid (PA), citric acid (CA), reduced/oxidized nicotinamide adenine dinucleotide (NADH/NAD^+^), succinate dehydrogenase (SDH), cytochrome c oxidase (CO), adenosine triphosphate (ATP), acetyl CoA, acetoacetate, and β-hydroxybutyric acid.

### 2.6. Data Analysis

Statistical analysis of enzyme indicators and related gene expression was performed using ANOVA in IBM SPSS Statistics 25 software, and *p* < 0.05 was considered as a specific statistical difference; correlation of genus-level rumen microorganisms (Top20) with mitochondrial enzyme indicators and genes was analyzed using Spearman analysis (R > 0.5); and correlation between metabolites and phenotypic values was analyzed using weighted gene co-expression network analysis (Weighted gene co-expression network analysis, WGCNA) [39] to analyze the correlation between metabolites and phenotypic values.

## 3. Results

### 3.1. Ultrastructure and Mitochondrial Dynamics of Rumen Epithelium in Tibetan Sheep at Different Altitudes

**Figure 2:**
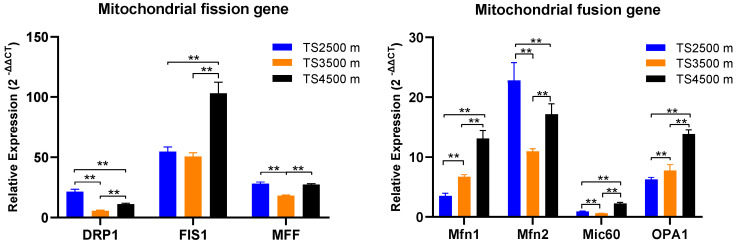
Expression of genes related to mitochondrial dynamics in the rumen epithelium of sheep at different altitudes. ** indicates extremely significant differences.

Ultrastructural observation of the rumen epithelium of Tibetan sheep at different altitudes revealed (Figure 1) that the tightness of the epithelial stratum corneum gradually increased with the elevation, the bridging granule linkages between the cells of the granular layer increased, the intercellular gaps narrowed, the number of mitochondria increased, and some of them appeared to be swollen and enlarged. A large number of mitochondria were found to be distributed in the sphenoidal layer and the basal lamina and they increased with the elevation. Compared with TS 2500 m, the area of mitochondria in the spiny layer at TS 3500 m and TS 4500 m increased, and the cellular gap in the spiny layer increased compared with the others. The number of mitochondria in the basal layer increased with elevation and some swelling of mitochondria was found in the basal layer of the rumen epithelium in the TS 3500 m and TS 4500 m groups. There was also an increase in intercellular bridging connections in the basal layer in the TS 4500 m group and the presence of hemidesmosomes in the basal membrane. Analysis of genes related to mitochondrial dynamics in the rumen epithelium at different altitudes revealed (Figure 2) that the expression of the splitting gene *FIS1* was the highest, being significantly higher in the rumen epithelium of the TS 4500 m group than in the other altitudes (*p* < 0.01), followed by the expression of *DRP1*. *MFF* in the TS 2500 m and TS 4500 m groups were significantly higher than that in the TS 3500m group (*p* < 0.01). Meanwhile, the four mitochondrial fusion genes had higher expression of *Mfn1*, *Mfn2*, and *OPA1*, among which the expression of *Mfn1* and *OPA1* increased with elevation, and the expression of *Mfn2* was significantly higher in the TS 2500 m and TS 4500 m groups than in the TS 3500m group (*p* < 0.01).

### 3.2. Functional Characteristics of Mitochondria in the Rumen Epithelium of Tibetan Sheep at Different Altitudes

**Figure 3:**
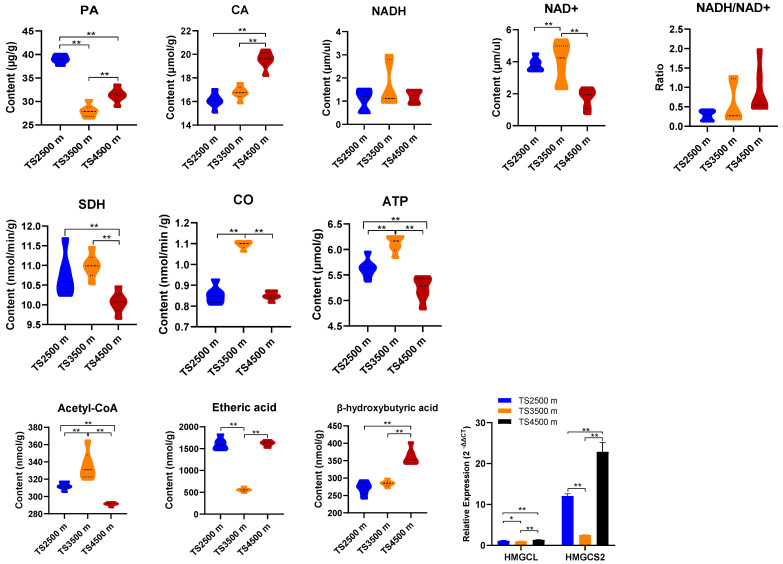
Analysis of key enzymes of energy metabolism in rumen epithelial mitochondria of Tibetan sheep at different altitudes. * indicates a significant difference, ** indicates a highly significant difference.

Analysis of key enzyme indices and related genes involved in mitochondrial energy metabolism processes (tricarboxylic acid cycle, oxidative phosphorylation, and ketogenesis) in the rumen epithelium of Tibetan sheep at different altitudes (Figure 3) revealed the following:

Tricarboxylic Acid Cycle: Pyruvate (PA) content in the rumen epithelium of the TS 3500m and TS 4500m groups was significantly reduced (*p* < 0.01). Citrate (CA) content increased with altitude elevation, reaching a significant peak in the TS 4500m group compared to other altitudes (*p* < 0.01). Oxidative Phosphorylation: NADH (Complex I) showed no significant differences across altitudes (*p* > 0.05). NAD^+^ levels were significantly higher at TS 3500m than in the other groups (*p* < 0.01) but significantly lower at TS 4500m (*p* < 0.01). The NADH/NAD^+^ ratio remained unchanged across altitudes (*p* > 0.05). Succinate dehydrogenase (SDH, Complex II) and cytochrome c oxidase (COX, Complex IV) activities were significantly reduced at TS 4500m (*p* < 0.01) but highest at TS 3500m. ATP content was lowest at TS 4500m and highest at TS 3500 m. Ketogenesis: Acetyl CoA, a key enzyme index in energy metabolism, was significantly reduced at TS 4500m (*p* < 0.01) but highest at TS 3500m. β-Hydroxybutyrate levels increased significantly with altitude (*p* < 0.01), while acetoacetate peaked in the TS 4500m and TS 2500m groups. The ketogenesis-related gene HMGCS2 exhibited higher expression than HMGCL, with TS 4500 m showing a significant upregulation compared to other altitudes (*p* < 0.01).

### 3.3. Analysis of Microflora–Mitochondrial Structure–Function Interactions

**Figure 4:**
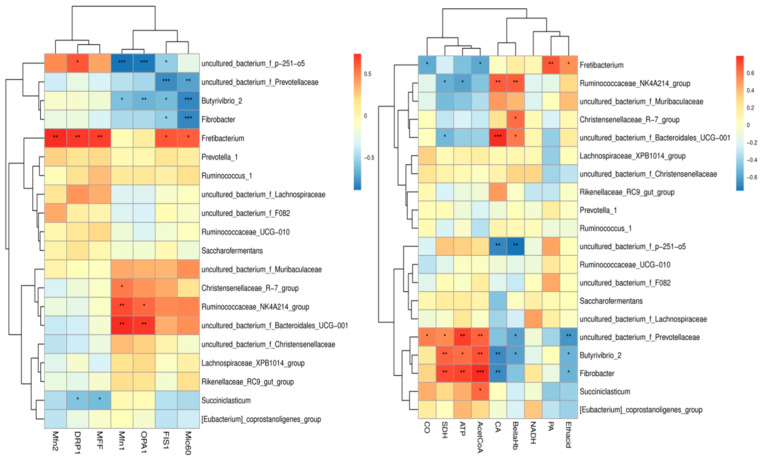
Microbiota–host gene interaction heatmap. * *p* < 0.05, ** *p* < 0.01, *** *p* < 0.001.

Based on the results of the existing analysis of the rumen microbiome of Tibetan sheep [40], the rumen microflora of Tibetan sheep at different altitudes were correlated with key enzyme indicators of mitochondrial energy metabolism processes and genes related to mitochondrial dynamics (Figure 4). Correlation heatmap analysis revealed that *Butyrivibrio_2*, *Fibrobacter*, and *uncultured_bacterium_f_Prevotellaceae* were significantly and positively correlated with acetyl CoA, ATP, and SDH (*p* < 0.05), and significantly and negatively correlated with CA, β-hydroxybutyrate, and acetoacetate (*p* < 0.05) *Ruminococcaceae_NK4A214_group* was significantly and positively correlated with CA and β-hydroxybutyrate (*p* < 0.05). Correlation analysis with genes related to mitochondrial dynamics revealed that *Butyrivibrio_2*, *Fibrobacter* and *uncultured_bacterium_f_ Prevotellaceae* were significantly negatively correlated with *Mic60* and *FIS1* (*p* < 0.05); *uncultured_bacterium_f_p-251-o5* was significantly negatively correlated with *Mfn1* and *OPA1* (*p* < 0.05); *Fretibacterium* was significantly and positively correlated with *MFF*, *DRP1*, and *Mfn2* (*p* < 0.05); and *Ruminococcaceae_NK4A214_group* and *uncultured_bacterium_f_Bacteroidales_UGG_001* were significantly and positively correlated (*p* < 0.05) with *Mfn1* and *OPA1*.

### 3.4. Analysis of Metabolite–Mitochondrial Structure–Function Interactions in Rumen Microbiota

**Figure 5:**
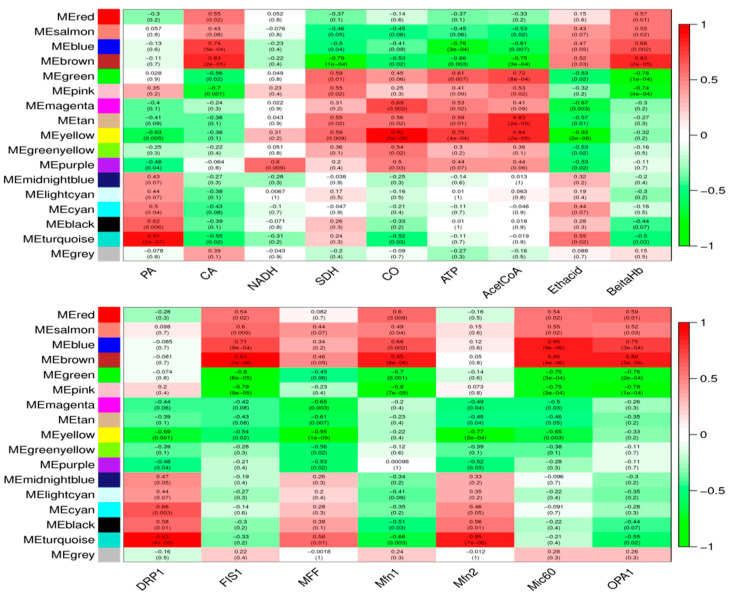
WGCNA of rumen microbial metabolites, key enzyme indexes, and mitochondrial dynamics-related genes of Tibetan sheep.

Weighted gene co-expression network analysis (WGCNA) of key enzyme indicators of mitochondrial energy metabolism process and genes related to mitochondrial kinetics with pre-metabolomics data [41] revealed that rumen microbial metabolites of Tibetan sheep at different altitudes were mainly classified into 17 modules, which were differently correlated with the key enzyme indicators of mitochondrial energy metabolism process and genes related to mitochondrial kinetics to a certain degree. These modules were differently correlated with key enzyme indicators of mitochondrial energy metabolism and genes related to mitochondrial dynamics (Figure 5). Among them, *FIS1*, *Mic60*, *Mfn1*, *OPA1*, CA, and β-hydroxybutyric acid were significantly and positively correlated with the MEbrown module, where the top 10 metabolites were TDP-glucose, 6-Phosphonoglucono-D-lactone, XDP, dTTP, 3-chloro-D-alanine, Oxalureate, D-allulose-6-phosphate, 7-Methylguanine, CDP, and L-Ascorbate 6-phosphate were significantly negatively correlated with MEgreen and MEpink modules (*p* < 0.05); *DRP1*, *Mfn2*, *MFF*, PA, and acetoacetic acid showed significant positive correlation (*p* < 0.05) with MEturquoise module, where the top 10 metabolites were Histidinyl-Aspartate, N2-hydroxyguanosine 5′-monophosphate, Pyrrolidonecarboxylicacid,3,3-Dimethyl-2-butanol, DG(16:1(9Z)/16:1(9Z)/0:0), 1,2-Dimethoxy-3-propylbenzene, distearoyl phosphatidate, 6′′-O-Acetylglycitin, FMNH, and bacimethrin, MEyellow showed a significant negative correlation (*p* < 0.05) with *DRP1*, *Mfn2*, *MFF*, and *Mic60*, and a significant positive correlation with SDH, cytochrome c oxidase CO, ATP, and acetyl CoA, as well as with metabolites such as N-Succinyl-L-glutamate, Tacrine, fructoselysine, 5′-azido-5-deoxyuridine, N,N-Dimethylsphingosine, Salidroside, dihomo-gamma-linolenic acid, Pyridoxine, Dihydrouracil, and Cyclizine.

## 4. Discussion

Due to the unique structure and function of the rumen epithelium in ruminants, this study analyzed the mechanisms of immunity and energy metabolism in the rumen epithelium of Tibetan sheep with respect to rumen microbial fermentation and its epithelial mitochondrial dynamics and functional interactions. The rumen is the most representative digestive organ in ruminants, and the rumen epithelium is a rich complex squamous flat epithelium consisting of a cuticle, granular layer, spicule layer, and basal layer, with a large number of mitochondria embedded in the spicule layer, which are involved in fatty acid and ketone metabolism [42]. The morphologic structure of the intact rumen epithelium is the structural basis for maintaining a normal rumen epithelial barrier function, and the barrier formed by the rumen epithelial cells, such as the stratum corneum and the stratum granulosum, is able to segregate harmful substances in the rumen to avoid harm to the organism [43]. The stratum spinosum is the intermediate between the granular layer and the basal layer, which contributes to the metabolism and transport of nutrients in the rumen epithelium [44]. Basal layer cells contain functionally intact mitochondria and other organelles that are primarily involved in rumen epithelial renewal and damage repair processes [45]; they are the cells in the rumen that contribute the most to the metabolic properties of the tissue (i.e., ketogenicity), and are therefore probably the most important rumen layer relative to energy metabolism in the animal as a whole [46]. In the rumen epithelium, the echinoderm layer and the granular layer are mainly attached to intercellular bridging junctions, which link the intercellular microfilaments of the echinoderm layer and the granular layer to form tight junctions between the epithelial cells. In the present study, ultrastructural observations of the rumen epithelium of Tibetan sheep grazing at different altitudes revealed an increase in the compactness of the stratum corneum, an increase in the number of bridging granule links between cells in the granular layer, an increase in the number of intercellular gaps, an increase in the number of mitochondria, and an increase in the distribution of mitochondria in the sphenoid layer and the basal lamina as a result of the increase in altitude. In the basal layer of the TS 4500m group, an increase in intercellular bridging granule linkages was found, as well as the presence of hemi-granules in the basal lamina. This is in agreement with the findings of Klevenhusen et al. [47], which showed that the rumen epithelium of Tibetan sheep has dense tight junctions and bridging granule junctions under high-altitude conditions, which are unique barriers used to regulate nutrient metabolism mechanisms related to the rumen epithelium under high altitude and low-oxygen conditions.

Mitochondrial division and fusion play a key role in maintaining mitochondrial function when cells undergo metabolic or environmental stress [48]. In this study, the mitochondrial kinetic analysis of rumen epithelium of Tibetan sheep at different altitudes revealed that the expression of the splitting gene *FIS1* was the highest, being significantly higher in the rumen epithelium of the TS 4500m group than in other altitudes (*p* < 0.01). This was consistent with Justin et al.’s [49] study, suggesting that mitochondria responded to metabolic demands under hypoxic conditions through the upregulation of *FIS1* and that the expression of *FIS1* may contribute to the maintenance of intracellular mitochondrial homeostasis. The expression of *DRP1* and *MFF* was significantly higher in the TS 2500m and TS 4500m groups than in the TS 3500m group (*p* < 0.01), suggesting that the enhancement of mitochondrial division under different altitudinal conditions, especially under hypoxic stress, may be a protective mechanism against the insufficient supply of oxygen by modulating the metabolic state of the mitochondria and the renewal rate to protect the function of key organs, including the gastrointestinal tract, and reduce potential damage, which helps to regulate the balance of energy production and consumption in organisms [50]. In mammals, fusion between outer mitochondrial membranes is mediated by the membrane-anchored dynamin family members *Mfn1* and *Mfn2*, whereas in mammals, fusion between inner mitochondrial membranes is mediated by a single dynamin family member, *OPA1*. The mitochondrial fusion genes *Mfn1*, *Mfn2*, and *OPA1* were higher in the present study, with *Mfn1* and *OPA1* expression increasing as elevation increases, while *Mfn2* was significantly higher in the TS 2500 m and TS 4500 m groups than in the TS 3500 m group (*p* < 0.01). This is because *Mfn1* and *OPA1* are core factors regulating mitochondrial fusion, which are indispensable in maintaining intracellular mitochondrial homeostasis. Under high-altitude conditions, the reduction in oxygen leads to impaired ATP synthesis, and in order to avoid excessive division of mitochondria, at this time, the cells adapt to the energy demand under hypoxic conditions by enhancing mitochondrial fusion in order to clear the damaged mitochondria and to maintain the dynamic equilibrium of the mitochondrial network [51].

Mitochondria are major loci for ATP production, calcium homeostasis and signaling regulation, and mediate apoptosis. Therefore, the structural and functional integrity of mitochondria is critical for the control of cellular health [52]. The TCA cycle is a key pathway for the metabolism of a variety of nutrients and is closely linked to cellular respiration, free radical production, and inflammatory responses [53]. Cell development requires the use of fatty acids to generate energy through oxidative phosphorylation, and fatty acid catabolism occurs in mitochondria, where fatty acids are converted to acetyl coenzyme A for further oxidation in the tricarboxylic acid cycle (TCA) [54]. As the electrochemical potential is transferred from the matrix to the intermembrane space via protons, the energy of the electrochemical potential is utilized by ATP synthase to drive the catalytic reaction ADP+ phosphate → ATP to satisfy the energy requirements for the cell to perform its functions [4], while oxidative phosphorylation is involved in the regulation of various key cellular activities such as calcium homeostasis, apoptosis, and cellular senescence [55]. The conversion of acetyl coenzyme A by the mitochondrial β-oxidation system produces reduced coenzyme I (NADH) in addition to ATP for energy [56]. Ketone bodies (mainly β-hydroxybutyric acid) produced by the rumen epithelium of ruminants not only provide energy to the rumen epithelial cells, but they also play a crucial role in the metabolic changes in the rumen epithelial cells [57]. In this study, pyruvate (PA) content in rumen epithelium of the TS 3500m and TS 4500 m groups was significantly decreased (*p* < 0.01). Pyruvate is a key substance in sugar metabolism and involved in the pyruvate–citrate cycle pathway for the transport of acetyl coenzyme A from mitochondria to the cytoplasm, and its decrease suggests that the metabolic pathway may be suppressed, whereas citric acid (CA) was increased with elevation and significantly higher in the rumen epithelium in the TS 4500 m group, being significantly higher than at other altitudes (*p* < 0.01), suggesting that mitochondria regulate tricarboxylic acid cycle homeostasis through the upregulation of citrate (CA) to maintain tissue health under low-oxygen conditions at high altitudes [58]. In addition, in the process of oxidative phosphorylation, NADH (complex I) did not differ significantly at different altitudes (*p* > 0.05), while the NAD^+^ content of Tibetan sheep in the TS 3500 m group was significantly higher than that at other altitudes (*p* < 0.01). Succinate dehydrogenase SDH (complex II) and cytochrome c oxidase CO (complex IV) were significantly lower in the TS 4500 m group (*p* < 0.01) and reached the highest in the TS 3500 m group. The analysis of ATP content measurements revealed that it was the lowest in the TS 4500 m group and the highest in the TS 3500 m. The results of the analysis showed that the energy metabolism process is a key enzyme in the energy metabolism process. Acetyl CoA was significantly reduced (*p* < 0.01) at TS 4500 m, while it was highest at TS 3500 m. This is because the core function of mitochondria is its ability to pump protons across the inner membrane in order to generate a gradient with high potential energy (i.e., the mitochondrial membrane potential), which is subsequently used to drive the synthesis of ATP by the multisubunit ATP synthase complex [59]. The decrease in redox potential and the obstruction of oxidative phosphorylation in mitochondria at high altitude with low oxygen, which prevents the normal synthesis of ATP, leads to a rapid decrease in ATP production. Thus, the mitochondrial oxidative phosphorylation process could proceed smoothly in the middle altitude group environment. The rumen microbial fermentation after ruminant dietary intake produces VFAs, in which butyric acid is oxidized to generate acetyl coenzyme A. Then, acetyl coenzyme A enters the mitochondria to oxidize and generate ketone bodies through a process of ketogenicity, in which the key enzyme of ketogenicity (*HMGCS2*), as a rate-limiting enzyme, has a greater influence on the generation of ketone bodies. The level of the activity of the enzyme of *HMGCS2* thus has a direct effect on the content of the carcasses of the organisms (mainly the β -hydroxybutyrate) [60]. NEWMAN et al. [61,62] reported that β-hydroxybutyric acid, as an important substance for the conversion and generation of volatile fatty acids in rumen epithelial cells, not only serves as an important energy substrate for the body to increase the energy demand but also acts as a signaling molecule. In this experiment, the content of β-hydroxybutyric acid increased significantly with increasing altitude (*p* < 0.01). The expression of the ketogenic pathway-related gene, *HMGCS2*, was significantly higher in the TS 4500 m group than in the other altitude groups (*p* < 0.01), which may be due to the significantly higher content of butyric acid in the rumen VFAs of Tibetan sheep in the high-altitude group than in the other altitudes.

As the “second genome” of animals, the GI microbiota plays an important role in the adaptation of animals to the highland environment [63]. *Fibrobacter* is the main fiber-digesting bacterium in the gastrointestinal tract of herbivores, especially in the rumen, and it is considered to be the main bacterial degrader of lignocellulosic material in the gut of herbivores [64]. *Butyrivibrio_2* and its availability in the gastrointestinal tract is essential for the physiological development of the host animal’s gastrointestinal tract; the maintenance of host health [65] should be related to diet and their availability in highland regions. This reviewer would surmise that animals in high-altitude areas consume slightly different proportions of lignocellulosic content of forages [66], and it has been found that the regulation of the gut microbiota has allowed for the *Ruminococcaceae_ NK4A214_Group* to influence the absorption of vitamin A in the host intestinal tract, which was significantly correlated with vitamin metabolism [67]. The content of Fibrobacter in the rumen of Tibetan sheep at high altitudes was significantly higher than that at other altitudes in this experiment (*p* < 0.05). Through correlation analysis, we found that *Butyrivibrio_2* and *Fibrobacter* showcased significant negative correlation with *Mic60* (*p* < 0.01), as well as significant positive correlation with acetyl CoA, ATP, and SDH (*p* < 0.01). *Ruminococcaceae_ NK4A214_Group* showed significant positive correlation (*p* < 0.05) with *Mfn1* and *OPA1*. Further WGCNA analysis of Tibetan sheep rumen microbial metabolites with key enzyme indicators of mitochondrial energy metabolism and mitochondrial kinetic genes revealed that the splitting gene *FIS1*, the fusion genes (*Mic60*, *Mfn1*, *OPA1*), mitochondrial energy metabolism-related enzymes CA, and β-hydroxybutyric acid were significantly and positively correlated with the MEbrown module. The MEyellow module was significantly and positively correlated with the splitting genes (*DRP1*, *MFF*), fusion genes (*Mfn2*, *Mic60*), and with enzymes involved in mitochondrial oxidative phosphorylation (SDH, cytochrome c oxidase CO, ATP, and acetyl CoA), suggesting that the rumen microbial metabolites, together with these genes and enzymes, play a role in mitochondrial energy metabolism to regulate the rumen of Tibetan sheep, as well as in microbial fermentation with its epithelial mitochondrial kinetic energy metabolism mechanism.

## 5. Conclusions

**Figure 6:**
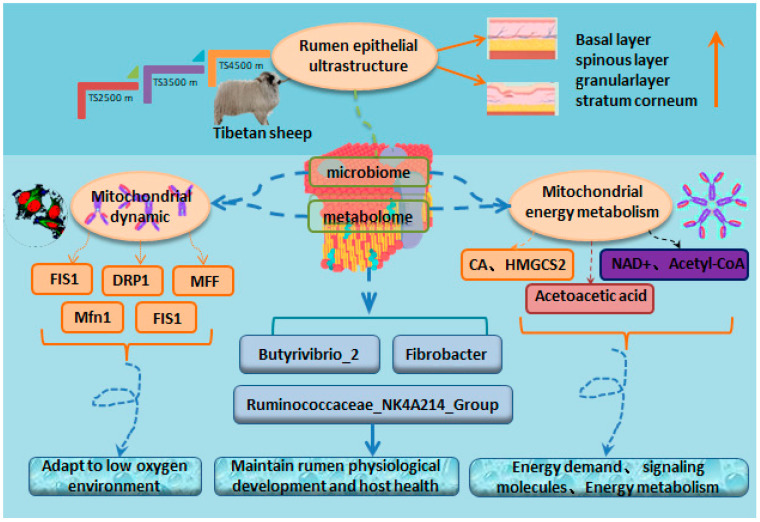
Machine Diagram.

The ultrastructure of the rumen epithelium of Tibetan sheep at different altitudes showed enhanced compactness of the stratum corneum, increased bridging granule links between cells in the granular layer, cellular gaps, number of mitochondria, and distribution of mitochondria in the stratum spinosum and basal lamina as the altitude increased. Split gene expression was higher in the rumen epithelium of the TS 4500m group than at other elevations, and the expression of the fusion genes *Mfn1* and *OPA1* increased with elevation. The analysis of mitochondrial energy metabolism showed that pyruvate content was significantly decreased in the TS 3500m and TS 4500m groups, and citrate increased with elevation; NAD^+^ content was significantly higher in TS 3500m Tibetan sheep than at other elevations, and was significantly decreased at TS 4500m; SDH, cytochrome c oxidase CO, and acetyl CoA reached the highest at TS 3500m; *β-hydroxybutyric* acid content increased significantly with elevation; *HMGCS2* expression was higher than *HMGCL* and showed significantly higher expression at TS 4500m than other elevations (*p* < 0.01). The correlation analysis between rumen microflora and mitochondrial energy metabolism showed that *Butyrivibrio_2* and *Fibrobacter* showed highly significant negative correlation with Mic60 and highly significant positive correlation with acetyl CoA, ATP, and SDH (*p* < 0.01). *Ruminococcaceae_NK4A214_Group* showed highly significant positive correlations with *Mfn1* and *OPA1* (*p* < 0.05). WGCNA found that the rumen microbial metabolites of Tibetan sheep at different altitudes were mainly classified into 17 modules, of which *DRP1*, *Mfn2*, *MFF*, PA, and acetoacetic acid were significantly positively correlated with the MEturquoise module (*p* < 0.05); MEyellow was significantly negatively correlated with *DRP1*, *Mfn2*, *MFF*, and *Mic60* (*p* < 0.05) (Figure 6).

## Figures and Tables

**Table 1:** Primer information.

Gene	Primer Sequence	Length/bp	Tm/°C	GenBank No
*β-Actin*	F:AGCCTTCCTTCCTGGGCATGGA	113	60	NM_001009784.3
	R:GGACAGCACCGTGTTGGCGTAGA			
*Drp1*	F: AGGAATGACCAAGGTGCCTG	148	60	XM_015094867.4
	R: AAGTGCCTCTGATGTTGCCA			
*Fis1*	F: TGAAGTATGTGCGAGGGCTG	108	60	XM_027961118.1
	R: CCATGCCCACTAGTCCATCTTT			
*MFF*	F: TCCAGCACGTGCATACTGAG R: CCGCCCCACTCACTAAATGT	107	60	XM_027965256.1
*Mfn1*	F: TGGGCATCATCGTTGTTGGA	137	60	XM_004003134.5
	R: AAAGGCTCTCTCCTTGGCAC			
*Mfn2*	F: ATGAACTGCACCGCCACATA	196	60	XM_004013714.5
	R: TTGAGGTCGTAGCTGAGGGA			
*OPA1*	F: ATCTTCCAGCTGCACAGACC	113	60	XM_012140446.1
	R: CCAAGCTACCTCGACTGCTT			
*Mic60*	F: TTGAGATGGTCCTTGGTT	136	60	XM_012169573.1
	R: TTGTTTCTGAGGTGGTGAG			
*HMGCS2*	F: GCCCTGGACAAATGTTACGC	132	60	XM_004002390.5
	R: GACCAACTTGCAGAAAGGCG			
*HMGCL*	F: CCAGCTTCGTGTCTCCCAAA	103	60	XM_004005125.4
	R: GGGGTCAGGACTGGGTAGTT			

## Data Availability

The datasets presented in this study can be found in online repositories. The names of the repository/repositories and accession numbers can be found below: (Sequence Read Archive (SRA): PRJNA818841 (Microbial sequence)/PRJNA819418 (Transcriptome sequence)).

## Abbreviations

*Drp1*	dynamin-related protein 1
*Fis1*	mitochondrial fission protein 1
*Mfn1*	mitofusin 1
VFAs	Volatile Fatty Acid
*HMGCS*	β-hydroxy-β-methylglutaryl-CoA synthase
*HMGCL*	β-hydroxy-β-methylglutaryl-CoA lyasee
